# Transcatheter Aortic Valve Implantation and Morbidity and Mortality-Related Factors: a 5-Year Experience in Brazil

**DOI:** 10.5935/abc.20160072

**Published:** 2016-06

**Authors:** André Luiz Silveira Souza, Constantino González Salgado, Ricardo Mourilhe-Rocha, Evandro Tinoco Mesquita, Luciana Cristina Lima Correia Lima, Nelson Durval Ferreira Gomes de Mattos, Arnaldo Rabischoffsky, Francisco Eduardo Sampaio Fagundes, Alexandre Siciliano Colafranceschi, Luiz Antonio Ferreira Carvalho

**Affiliations:** 1 Hospital Pró-Cardíaco, Rio de Janeiro, RJ - Brazil; 2 Pós-graduação em Ciências Cardiovasculares - Universidade Federal Fluminense, Rio de Janeiro, RJ - Brazil; 3 Pós-graduação em Ciências Médicas - Universidade do Estado do Rio de Janeiro, Rio de Janeiro, RJ - Brazil

**Keywords:** Aortic Valve Stenosis / surgery, Mortality, Prosthesis Implantation, Balloon Valvuloplasty, Cohort Studies

## Abstract

**Background:**

Transcatheter aortic valve implantation has become an option for high-surgical-risk patients with aortic valve disease.

**Objective:**

To evaluate the in-hospital and one-year follow-up outcomes of transcatheter aortic valve implantation.

**Methods:**

Prospective cohort study of transcatheter aortic valve implantation cases from July 2009 to February 2015. Analysis of clinical and procedural variables, correlating them with in-hospital and one-year mortality.

**Results:**

A total of 136 patients with a mean age of 83 years (80-87) underwent heart valve implantation; of these, 49% were women, 131 (96.3%) had aortic stenosis, one (0.7%) had aortic regurgitation and four (2.9%) had prosthetic valve dysfunction. NYHA functional class was III or IV in 129 cases (94.8%). The baseline orifice area was 0.67 ± 0.17 cm^2^ and the mean left ventricular-aortic pressure gradient was 47.3±18.2 mmHg, with an STS score of 9.3% (4.8%-22.3%). The prostheses implanted were self-expanding in 97% of cases. Perioperative mortality was 1.5%; 30-day mortality, 5.9%; in-hospital mortality, 8.1%; and one-year mortality, 15.5%. Blood transfusion (relative risk of 54; p = 0.0003) and pulmonary arterial hypertension (relative risk of 5.3; p = 0.036) were predictive of in-hospital mortality. Peak C-reactive protein (relative risk of 1.8; p = 0.013) and blood transfusion (relative risk of 8.3; p = 0.0009) were predictive of 1-year mortality. At 30 days, 97% of patients were in NYHA functional class I/II; at one year, this figure reached 96%.

**Conclusion:**

Transcatheter aortic valve implantation was performed with a high success rate and low mortality. Blood transfusion was associated with higher in-hospital and one-year mortality. Peak C-reactive protein was associated with one-year mortality.

## Introduction

Transcatheter aortic valve implantation (TAVI) was first introduced in 2002 as an alternative treatment for patients with aortic stenosis (AoS) at extreme risk for surgery.^1^ As from 2008, it has become available in Brazil, in parallel with major technological advances and the publication of large-scale randomized clinical trials demonstrating the benefits of this treatment in the relief of symptoms as well as in mortality reduction.^2-6^ TAVI indications also expanded to the treatment of bioprosthetic valve dysfunction and selected cases of aortic regurgitation.

In order to make concepts uniform and establish comparable parameters, a group of highly regarded authors, under the name of Valve Academic Research Consortium (VARC), proposed procedure-related criteria of success and complications.^7,8^ Despite its importance, many authors were initially reluctant to adhere to the VARC criteria, possibly because their strict concepts could lead to the perception of unfavorable results.

The first case series of TAVI in the State of Rio de Janeiro was published in 2010,^9^ and these patients as well as the progression of the technique have been followed up ever since, thus adjusting the assessments to the VARC criteria. In this study, we evaluated the success rate, morbidity and mortality throughout in-hospital and one-year follow-up in a 5-year-experiece with TAVI.

## Methods

### study population

Prospective cohort study of consecutive cases of TAVI between July 2009 and February 2015. TAVI was indicated for patients with severe heart valve stenosis, severe aortic regurgitation or bioprosthetic aortic valve dysfunction at a high surgical risk. Demographic, echocardiographic, laboratory and procedural data were assessed during the in-hospital and one-year outpatient follow-up. Heart failure symptoms were classified according to the New York Heart Association (NYHA) criteria, and the success and complications criteria were based on VARC 2: hospital discharge, aortic regurgitation < grade 2/4, mean left ventricular-aortic pressure gradient (LV-Ao) < 20 mmHg, and use of only one prosthesis. Definitions of complications by VARC are described elsewhere, and include criteria of acute myocardial infarction (AMI) and stroke^7^. Chronic renal failure (CRF) was defined as an estimated glomerular filtration rate < 60 mL/minute using the Cockroft and Gault formula. Acute renal failure (ARF) was defined by the Acute Kidney Injury Network (AKIN) classification system as follows: stage 1, if serum creatinine (Cr) elevation between 1.5 and 1.99 times; stage 2, if between 2 and 2.99 times; and stage 3, if greater than 3 times or need for dialytical support.^7^

### Pre-procedural assessment

Indications were evaluated by the cardiology team, which was comprised of cardiology clinicians, interventionists, surgeons, anesthetists, and echocardiographers. Once TAVI was indicated, all patients would undergo coronary angiography and assessment of coronary artery disease, with occasional indication of coronary angioplasty, which was left to the discretion of the surgeon. Aorta and iliac branches were measured by angiography and/or CT angiography to define the prostheses and vascular approach to be used.

### Procedural technique

All patients received antibiotic prophylaxis with cefazolin 2 g prior to the intervention. Acetylsalicylic acid (ASA) 200 mg and clopidogrel 300 mg were administered on the day before the procedure, except when contraindicated in the cases of low platelet count < 80 x 10^3^ /mm^3^ or other comorbidities. The procedures were performed in the hemodynamics laboratory or in the hybrid room, under sedation or general anesthesia, and transesophageal echocardiography (TEE) monitoring. Temporary transvenous pacemakers were implanted to help in balloon valvuloplasty and/or prosthesis implantation, by means of induction of tachycardia, and were kept on the on-demand mode for 48 hours. The choice of of whether or not to pre-dilate the valve was left to the surgeon's discretion. The prostheses used were the self-expanding CoreValve^®^ (Medtronic, Minneapolis, MN) and the balloon-expanding Edwards-SAPIEN XT^®^ (Edwards Lifesciences, Irvine, CA). After TAVI, the patients were sent to the intensive care unit and underwent daily laboratory assessments in the first 7 days.

### Late follow-up

The outpatient follow-up was performed via phone calls at 30 days, 6 months and 1 year, and clinical, echocardiographic and adverse events data were recorded.

The study was approved by the local Research Ethics Committee under registration 423, on April 8, 2011. All patients gave written informed consent to participate in the study.

### Statistical analysis

Continuous variables were expressed as mean and standard deviation, for parametric variables; or median and interquartile range for non-parametric variables. Categorical variables were expressed as absolute and percentage values. Numerical data were compared using the *t* test for parametric variables, and the Mann-Whitney test for non-parametric variables. The chi square test or Fisher's exact test were used to compare proportions. The Kaplan-Meier method was used to adjust the 1-year survival curve. The significance level was set at 5%. Logistic regression analysis was carried out to evaluate the simultaneous influence of different variables, by means of the stepwise forward analysis, at a significance level of 5%, selecting the smallest subgroup of independent variables able to better predict death. The statistical analysis was processed by the SAS^®^ version 6.11 statistical software (SAS Institute, Inc., Cary, North Carolina) and Statistical Package for the Social Sciences (SPSS), version 18.0.

## Results

TAVI was performed in 136 patients with a mean age of 83 years (80 to 87); 49.3% were women (Table 1). The indications were as follows: 131 (96.3%) patients with AoS, one (0.7%) with aortic regurgitation, and four (2.9%) with bioprosthetic aortic valve dysfunction. The risk of surgical mortality using the Surgeons Thoracic Society (STS) score was 9.3% (4.8-22.3%), and an STS ≥ 15% was observed in 39.8% of cases.

**Table 1:** Demographics

				n = 136
Age				83 (80-87)
Female gender				67 (49.3%)
BMI				25.3 (22.6-29-4)
Presentation				
	Syncope			40 (29.4%)
	Angina pectoris			28 (20.6%)
	Heart failure			
	NYHA functional class			
			II	7 (5.1%)
			III	71 (52.2%)
			IV	58 (42.6%)
Systemic hypertension				80 (67.2%)
Diabetes mellitus				51 (37.5%)
Hypercholesterolemia				65 (47.8%)
Previous AMI				17 (12.5%)
Coronary artery disease				77 (56.6%)
Previous CABG				30 (25.2%)
Previous PCI, days				46 (33.8%)
			> 30	29 (21.3%)
			< 30	17 (12.5%)
Previous stroke				8 (5.9%)
Peripheral vascular disease				32 (23.5%)
COPD				13 (9.6%)
Chronic kidney failure				70 (51.5%)
Pulmonary arterial hypertension				33 (24.3%)
Sinus rhythm				102 (75%)
Permanent atrial fibrillation				14 (10.3%)
Previous pacemaker				19 (14.7%)
Logistic euroSCORE (%)				19.1 (11.4-31.1)
STS mortality (%)				9.3 (4.8-22.3)
EF< 50%				36 (26.5%)

BMI: body mass index; NYHA: New York Heart Association; AMI: acute myocardial infarction; CABG: coronary artery bypass grafting; PCI: percutaneous coronary intervention; COPD: chronic obstructive pulmonary disease; STS: Surgeons Thoracic Society; EF: ejection fraction.

Other comorbidities were hypothyroidism (18.4%), previous malignancy (8.1%); asthma (5.9%); hepatic cirrhosis (2.2%); digestive hemorrhage (2.2%); porcelain aorta (2.2%); abdominal aortic aneurysm (4.4%); previous aortic balloon valvuloplasty (3.7%); and previous alcohol septal ablation (1.5%).

The baseline laboratory tests showed: type-B Brain Natriuretic Peptide (BNP) of 258 pg/mL (128 to 616 pg/mL), and greater than 200 pg/mL in 40.0%; Cr 1.2±0.8 mg/dL; platelets 194 x 10^3^ /mm^3^ (156 to 236 x 10^3^ /mm^3^); and hemoglobin 11.8 mg/dL (10.4 to 13.1 mg/dL). Baseline C-reactive protein (CRP) was elevated (> 0.3 mg/dL) in 57.8% of cases. Medications used by the patients are shown in Table 2. Blood transfusion prior to the procedure was made in eight patients (5.8%).

**Table 2:** Medications used prior to transcatheter aortic valve implantation

	n = 136
ACEI/ARB	63 (46.23%)
Betablocker	47 (34.6%)
Calcium antagonist	30 (22.2%)
Nitrates	13 (9.6%)
Diuretics	66 (48.5%)
Digitalis	8 (5.9%)
Coumarin	7 (5.1%)
Antiarrhythmic drugs	25 (18.4%)
Statins	77 (56.6%)
Vasoactive drugs	4 (2.9%)

ACEI: angiotensin-converting-enzyme inhibitor; ARB: angiotensin receptor blocker.

Findings of the baseline TEE are shown in Table 3. Ejection fraction (EF) < 50% was found in 26.5% of patients, and bicuspid aortic valve, in 2.9%. A mean LV-Ao gradient < 40 mmHg was found in 46/131 cases (35.1%).

**Table 3:** Baseline echocardiogram

		n = 136
AVA		0.67 ± 0.17
Peak LV-Ao gradient (mmHg)		78.8 ± 29.5
Mean LV-Ao gradient (mmHg)		47.3 ±18.2
Aortic regurgitation		
	Absent	43 (31.6%)
	Mild	76 (55.9%)
	Moderate	11 (8.1%)
	Severe	6 (4.4%)
Mitral regurgitation		
	Absent	19 (14.0%)
	Mild	89 (65.4%)
	Moderate	20 (14.7%)
	Severe	8 (5.9%)
EF (%)		59.5 ± 17.0
LV end-diastolic diameter (mm)		50.6 ± 10.5
Interventricular septum (mm)		12.0 ± 2.3
Upper wall (mm)		11.9 ± 2.1
PASP (mmHg)		44.1 ± 14.4

AVA: aortic valve area; LV-Ao: left ventricular-aortic; EF: ejection fraction; PASP: pulmonary artery systolic pressure.

In addition to angiography of the iliac and femoral arteries, CT angiography of these arteries was also performed in 17.6% of individuals. Coronary percutaneous interventions were performed prior to TAVI in eight patients (5.9%) and peripheral percutaneous interventions in four (2.95%) (one in carotid, two in iliac and one in subclavian artery).

The first 29 procedures (21.3%) were performed under sedation, and all the subsequent 107 (78.7%), under general anesthesia - in these cases, always monitored by TEE. A total of 52 procedures (38.2%) were performed in a hybrid room, as from March 2013.

The vascular access was the femoral artery in 129 cases (94.9%), left subclavian artery in six (4.4%), and aorta in one (0.8%). All vascular accesses were made via arteriotomy and further surgical vascular suture. A hemostasis device was used in only one case.

Heart valve pre-dilatation was performed in 110 patients (80.9%) and direct implantation, in 26 (19.1%). The self-expanding CoreValve^®^ prosthesis was implanted in 132 patients (97%) and the balloon-expanding Edwards SAPIEN XT^®^ prosthesis, in four (3%).

VARC2 success was achieved in 83.1% of cases. After TAVI, the invasive LV-Ao gradient dropped from 54.8 ± 25.5 mmHg to 1.7 ± 3.4 mmHg (p < 0.001). An additional intervention for correction of paraprosthetic aortic valve regurgitation was required in 55 cases (40.4%); balloon post-dilatation in 48 (35.5%); additional prosthesis implantation in six (4.4%); and prosthesis repositioning by loop traction in one (0.7%). Post-TAVI aortic regurgitation was considered absent in 53 patients (39%), mild in 71 (52.2%) and moderate in eight (5.9%) - all due to paraprosthetic regurgitation.

ARF occurred in 15.4% of patients, and 2.2 reached stage 3. The volume of contrast medium used was 143.0 ± 37.1 mL. There was one case of ischemic stroke with no sequela. There was no procedure-related AMI.

New permanent pacemaker implantation was required in 29/118 cases (24.5%).

The blood transfusion rate after TAVI was 21.3% (29 patients), of which eight received transfusion of two or three units of red blood cell concentrate, and ten received four or more. Perioperative bleeding related to the vascular access occurred in three cases; however, blood transfusions were performed for other complications such as LV perforation and hemothorax.

The length of hospital stay was 7 ± 22 days. Prolonged hospital stay (> 7 days) occurred in 51/125 cases (40.8%), with a maximum of 212 days.

Perioperative mortality rate was 1.5%; 30-day mortality was 5.9%, and in-hospital mortality was 8.1%. When the subgroup of in-hospital death was compared to that of patients discharged, we observed that the first showed higher baseline BNP [770 pg/mL (320-1.260) vs 227 pg/mL (123-553); p = 0.017]; a higher incidence of pulmonary arterial hypertension (54.6% vs 21.6%; p = 0.024); CRF (81.8% vs 51.2%; p=0.048); and ARF (45.5% vs. 11.2%; p = 0.008). Post-dilatation (70% vs. 35.2%; p = 0.034) and blood transfusion after TAVI (90.9% vs. 17.1%; p < 0.0001) were also more frequent. In the first week, there was higher peak CRP [13.1 mg/dL (6.8-17.5) vs 7.8 mg/dL (4.7-11.0); p = 0.039] and lower platelet count [99 x 10^3^ /mm^3^ (71-128) vs 143 x 10^3^ /mm^3^ (105-167); p = 0.030] among in-hospital death patients (Table 4). After logistic regression analysis, blood transfusion after TAVI (p = 0.0003) and pulmonary arterial hypertension (p = 0,036) were identified as independent predictors of in-hospital death (Table 5).

**Table 4:** Variables related to in-hospital and one-year mortality

		IH Death (n = 11)	Alive IH (n = 125)	p value	1-year death (n = 20)	Alive 1-year (n = 89)	p value
Age, years		84 (84-86)	83 (80-87)	0.28	84 (80-88)	83 (80-87)	0.43
Female gender		45.5%	49.6%	0.52	60.0%	48.3%	0.24
BMI (kg/m^2^)		24.7 (23-5-28.3)	25.3 (22.4-29.6)	0.65	24.9 (23.5-27.3)	25.3 (22.9-30.2)	0.81
NYHA class							
	II	36.4%	53.6%	0.28	50.0%	57.3%	0.52
	IV	54.6%	41.6%		50.0%	37.1%	
SH		72.7%	70.4%	0.59	55.0%	68.5%	0.19
DM		54.6%	36.0%	0.19	54.6%	36.0%	0.19
CAD		63.6%	56.0%	0.44	55.0%	53.9%	0.57
Previous CABG		27.3%	25.6%	0.57	30.0%	23.6%	0.37
Previous PCI		27.3%	34.4%	0.45	30.0%	33.7%	0.49
PVD		9.1%	24.8%	0.22	20.0%	20.2%	0.62
COPD		18.2%	8.8%	0.28	30.0%	6.7%	0.008
CRF		81.8%	51.2%	0.048	60.0%	47.2	0.22
PAH		54.6%	21.6%	0.024	40.0%	22.5%	0.09
euroSCORE		31.2 (12.6-52.2)	18.7 (11.2-30.7)	0.09	31.2 (16.3-42.0)	19.9 (10.4-28.2)	0.006
STS score		14.2 (6.5-30.5)	9.3 (4.8-20.8)	0.25	22.6 (11.9-36.2)	7.9 (4.4-19.6)	0.0005
EF < 50% (TEE)		36.4%	25.8%	0.33	40.0%	24.7%	0.14
Baseline Cr (mg/dL)		1.3 (0.7-1.7)	1.1 (0.9-1.50)	0.59	1.3 (0.9-1.4)	1.1 (0.9-1.3)	0.46
Baseline Hb (mg/dL)		11.4 (10.2-12.9)	11.8 (10.4-13.10	0.48	11.8 (9.0-12.9)	11.8 (10.7-13.3)	0.46
Pre platelets (x 10^3^/mm^3^)		189 (127-250)	195 (158-236)	0.53	218 (148-247)	193 (163-237)	0.49
Baseline BNP (pg/mL)		770 (320-1260)	227 (123-553)	0.017	536 (149-836)	230 (121-519)	0.065
Baseline CRP (mg/dL)		1.8 (0.2-5.5)	0.3 (0.2-1.0)	0.96	1.7 (0.2-2.3)	0.3 (0.2-1)	0.01
General anesthesia		90.9%	77.6%	0.27	55.0%	78.7%	0.032
TEE		81.8%	77.6%	0.54	50.0%	77.5%	0.016
Direct TAVI		36.4%	17.6%	0.13	40.0%	15.7%	0.02
Post-dilatation		70.0%	35.2%	0.034	47.4%	15.7%	0.15
AoR ≥ 2/4		12.5%	5.5%	0.40	15.0%	4.6%	0.12
Blood transfusion post		90.9%	17.1%	< 0.0001	60.0%	16.9%	0.0002
Cr in 72 hours (mg/dL)		1.9 (1.1-3.4)	1.2 (0.9-1.5)	0.06	1.3 (1.0-2.3)	1.2 (0.9-1.5)	0.16
ARF		45.5%	11.2%	0.008	30.0%	12.4%	0.058
Nadir Hb (mg/dL)		8.1 (7.5-11.4)	9.6 (8.2-10.9)	0.32	8.1 (7.4-11.2)	9.6 (8.3-10.9)	0.13
Platelets post (x 10^3^/mm^3^)		99 (71-128)	143 (105-167)	0.03	125 (73-175)	143 (106-167)	0.29
Peak CRP (mg/dL)		13.1 96.6-17.5)	7.8 (4.7-11.0)	0.04	13.1 (8.2-16-2)	7.5 (4.4-10.6)	0.001

IH: in-hospital; BMI: body mass index; NYHA, New York Heart Association; SH: systemic hypertension; DM: diabetes mellitus; CAD: coronary artery disease; CABG: coronary artery bypass grafting; PCI: percutaneous coronary intervention; PVD: peripheral vascular disease; COPD: chronic obstructive pulmonary disease; CRF: chronic renal failure; PAH: pulmonary arterial hypertension; STS: Surgeons Thoracic Society; EF: ejection fraction; Cr: serum creatinine; Hb: hemoglobin; BNP: type-B brain natriuretic peptide; CRP: C-reactive protein; TEE: transesophageal echocardiogram; TAVI: transcatheter aortic valve implantation; AoR: aortic regurgitation; ARF: acute renal failure.

**Table 5:** Logistic regression for in-hospital and one-year death

Significant variable	Coefficient	SE	p value	RR	95%CI
In-hospital death					
Blood transfusion post	3.9959	1.1075	0.0003	54.4	6.20-477
PAH	1.6666	0.7943	0.036	5.29	1.12-25.1
1-year death					
Blood transfusion post	2.1113	0.6367	0.0009	8.26	2.37-28.8
Peak CRP	0.1361	0.0550	0.13	1.15	1.03-1.28

SE: standard error; RR: relative risk; 95%CI: 95% confidence interval; PAH: pulmonary arterial hypertension; CRP: C-reactive protein.

**Figure 1:**
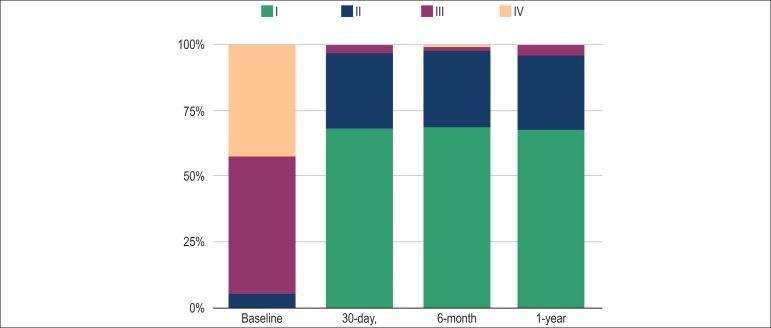
Baseline, 30-day, 6-month, and 1-year NYHA functional class. NYHA = New York Heart Association.

The follow-up lasted 2.5 ± 1.4 years. Progression of symptoms according to NYHA functional classes is shown in Figure 1.

**Figure 2:**
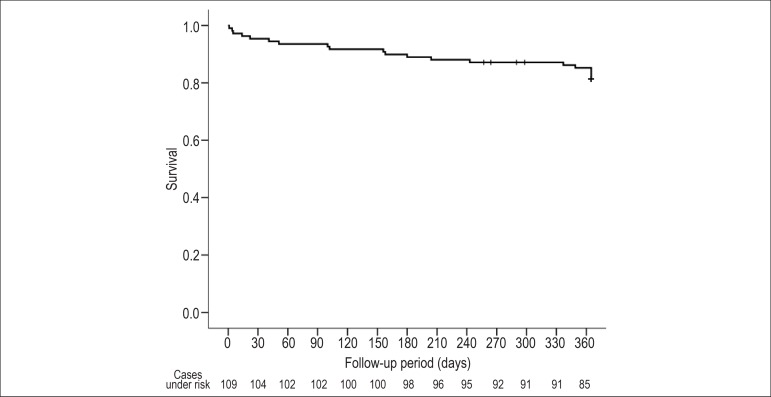
One-year survival Kaplan-Meier curve.

Accumulated overall one-year mortality was 18.3% (20/109) (Figure 2), of which cardiovascular mortality accounted for seven cases (two sudden deaths, one AMI for stent thrombosis, one for heart failure, two for hemorrhagic stroke and one for LV perforation When the subgroup of one-year death was compared to the group of survivors, we observed that the first group showed, among the pre-procedural characteristics, higher rates of chronic obstructive pulmonary disease (30% vs 6.7%; p = 0.008); logistic euroSCORE [31% (16-42) vs 19% (10-28); p = 0.006]; STS score [22% (12-36) vs 8% (4-19); p = 0.0005]; baseline CRP [1.7 mg/dL (0.2-2.3) vs 0.30 mg/dL (0.2-1.0); p = 0.01]; direct TAVI (40% vs 15.7%; p = 0.02); post-TAVI blood transfusion (60% vs 16.9%; p=0.0002); peak CRP [13.1 mg/dL (8.2-16.2) vs 7.5 mg/dL (4.4-10.6); p = 0.001]; and lower rates of general anesthesia (55% vs 78.7%; p = 0.032) and TEE (50% vs 77.5%; p=0.016). According to the logistic regression analysis, post-TAVI blood transfusion (p = 0.0009) and peak CRP (p = 0.013) were independent predictors of one-year death (Table 5).

## Discussion

This article describes the 5-year experience on self-expanding prosthetic heart valve implantation via femoral artery by means of arteriotomy in a medical center that has one of the highest case series in Brazil. Throughout this period, important conceptual changes had the following consequences: (1) lower tolerance to the presence of aortic regurgitation after the procedure; (2) adoption of general anesthesia associated with TEE monitoring, which enabled a more accurate quantification of the degree of paraprosthetic regurgitation and assessment of structural complications; (3) performance of procedures in a hybrid room; and (4) formalization of a team of cardiology specialists to share decision making.

The population characteristics are not different from those presented in most of the registries,^10-14^ including the national registry.^15^

Procedural success by VARC2 criteria achieved 83.1% in our cohort, whereas in the national registry it reached 76.3%. Currently, the literature demands considerable attention to the definitions adopted in the short-term results. The VARC2 criteria for device-implantation success include a mean transprosthetic gradient < 20 mmHg, absent or mild aortic regurgitation, and single-prosthesis implantation. As an example, Thyregod et at.^16^ recently reported to have adopted VARC2 criteria and found a 97.9% procedural success rate, although they had described the presence of moderate aortic regurgitation in 14.5% of cases, which would reduce the success rate to 83.4%.

The finding of an overall 30-day mortality of 5.9% in a group of very severely ill patients (mean STS of 15%) is a relevant fact. Registries from other countries showed 30-day mortality rates ranging from 5.2% to 10%,^10-12^ with 9.1% in the Brazilian national registry.^15^ Likewise, we should take care when comparing these results, once the VARC2 criteria recommend the description of in-hospital mortality, and not of 30-day mortality. In this case-series, this variation implied a 2.2% absolute increase, because three cases showed clinical complications that resulted in multiple organ failure and death after 1 month. To better understand the in-hospital course, in keeping with VARC updating, this short-term analysis was carried out using in-hospital mortality, while investigating in-hospital and one-year mortality-associated variables.

Independent factors associated with in-hospital mortality were the presence of pulmonary arterial hypertension and post-TAVI blood transfusion. Pulmonary arterial hypertension is one of the clinical risk factors for early death, regardless of procedural complications^17,18^ like CRF,^19,20^ which, in this analysis, was associated with early mortality. Blood transfusion was the most important independent variable for in-hospital mortality, although no distinction was made regarding its indication. We could speculate that this is a marker of severity common to three clinical situations: long intensive care unit stay,^21^ previous anemia followed by minor bleeding,^22-24^ or significant perioperative bleeding.^25^

The one-year accumulated overall mortality was 18.3%, one third of which was cardiovascular mortality. The independent risk factors for one-year mortality were post-TAVI blood transfusion and peak CRP. Escarcega et al. reported 37% of blood transfusion and, in additon to the increase in in-hospital mortality, they also verified an increase in one-year mortality in this subgroup (28% vs 13%; p = 0001).^22^

Like in coronary interventions, in TAVI there is a complex association between vascular complications, bleeding and blood transfusion, with the development of ARF and Systemic Inflammatory Response Syndrome (SIRS) - the latter being able to occur in a disproportionate fashion in relation to the triggering events described. Sinning et al.^26^ described that SIRS occurred in 40.1% of TAVI cases and was associated with higher 30-day mortality in addition to being an independent predictor of one-year mortality (hazard ratio - HR = 4.3; p < 0.001). The biomarker most frequently used in clinical practice for the assessment of SIRS is CRP, with a peak around day 3 after TAVI.^27^ Peak CRP in the in-hospital death subgroup was twice higher than that found among survivors.

The access was exclusively surgical, aiming to minimize vascular complications and bleedings. However, Bernardi et al.,^28^ compared the percutaneous and surgical accesses and did not identify differences between vascular complications, bleeding, 30-day mortality or one-year mortality, although they had found a tendency toward a higher frequency of peripheral vascular disease in the surgical access group (16.8% vs10.4%; p=0.07).^28^ In the national registry, the percutaneous access was finalized with a hemostasis device in 45.6% of cases.^15^

After the first 30 cases, the anesthetic regimen was changed from sedation to general anesthesia, incorporating three-dimensional TEE to the procedure. With this strategy, we aimed to measure the valve annulus in the procedure room, instead of previously using CT angiography, thus having the benefit of reducing nephrotoxicity. This strategy permits a thorough assessment of the degree of paraprosthetic regurgitation. In the Brazilian registry, as well as in our cohort, the use of TEE was associated with lower mortality,^14^ although this finding could merely have reflected our learning curve.

When the valve implantation technique was assessed, we observed that the pre-dilatation rate of 78.9% among our cases was higher than the 61% of the national registry.^14^ Our perception is that there was a tendency of direct implantation in the more severe cases to avoid the pacemaker-induced tachycardia maneuver.

The prognostic impact of moderate or severe paraprosthetic aortic regurgitation was a concept adopted as from 2011.^8^ Only 5.9% of cases of the present study showed this type of post-procedural complication, whereas other studies with predominance of self-expanding valves described rates between 10% and 15%.^4,16,29^ The fact that intervention was made in 40% of cases shows an aggressive management of paraprosthetic aortic regurgitation when compared to 16.1% to 26.5% of interventions described by other authors.^23,30^ Maybe for this reason, post-TAVI moderate aortic regurgitation was not predictive of poor prognosis in this study. However, we cannot fail to mention that post-dilatation was more frequent in the in-hospital death group, even with no directly related complications having been identified. This finding could reflect a selection bias of a subgroup with a less favorable anatomy. Alternatively, we should remember that pre- and post-balloon dilatation are performed under pacemaker-induced tachycardia, and this leads to systemic hypoperfusion, which, in turn, had been previously related to SIRS.^26^

The incidence of ARF was lower in comparison to a mean of 20% of other case series.^20,30^ Sinning et al^29)^ observ​ed that, in patients​ ​undergoing​ CoreValve® ​implantation, ERF correlated with peripheral vascular disease, SIRS ​and residual aortic regurgitation, but not directly with volume of contrast medium. Nuis et al.^31^ observed that the number of blood transfusions within the first 24 hours is the main risk factor for ARF, which also correlated with peripheral vascular disease, heart failure, and leukocytosis within the first 72 hours. Thus, ARF seems to correlate with hemodynamic instability, especially in the context of bleeding and blood transfusion, with further SIRS.

The virtual absence of stroke during hospital stay was much lower than the 4 to 5% described in other studies.^3,32^ We should point out that, in our protocol, antiplatelet agents are previously administered, and a careful technique is observed in the manipulation of the valve with the guidewire, in addition to a strict control of heparinization.

Management of coronary artery disease is another key issue, because it is present in half the cases of AoS. The extent of myocardial infarction and its possible relation to ventricular dysfunction suggests that it plays a role in the outcome and, currently, revascularization strategies are controversial.^33^ The strategy adopted was to perform revascularization in the cases in which a large ischemic area had been estimated by coronary angiography. In this cohort, there was no case of procedure-related AMI, which may suggest that this is an adequate strategy.

## Limitations

This study reflected the real-life practice, with inclusion of patients that would have been otherwise excluded in randomized studies. Because it is a prospective cohort, a selection bias cannot be ruled out. Despite the significant number considering the national figures, our case series is small compared to international registries in which predictors of poor prognosis were identified. Additionally, because of the sample size, some variables such as ARF stage or blood transfusion volume could not be stratified, with the purpose of more accurately predicting adverse events. The follow-up by phone calls made it difficult to have a consistent late outpatient assessment of aortic regurgitation and ventricular dysfunction.

## Conclusion

Transcatheter aortic valve implantation in patients with severe aortic valve disease and at high surgical risk was performed with a high success rate and low mortality. Relief of symptoms and one-year survival were high despite the severity of disease. Blood transfusion was associated with in-hospital and one-year mortality. Peak C-reactive protein was associated with one-year mortality.
